# Cancer specific promoter CpG Islands hypermethylation of HOP homeobox (HOPX) gene and its potential tumor suppressive role in pancreatic carcinogenesis

**DOI:** 10.1186/1471-2407-12-397

**Published:** 2012-09-07

**Authors:** Mina Waraya, Keishi Yamashita, Hiroshi Katoh, Akira Ooki, Hiroshi Kawamata, Hiroshi Nishimiya, Kazunori Nakamura, Akira Ema, Masahiko Watanabe

**Affiliations:** 1 Department of Surgery, Kitasato University Hospital, 1-15-1 Kitasato, Minami-ku, Sagamihara, Kanagawa, 252-0375, Japan

**Keywords:** HOP homeobox, Pancreatic cancer, Methylation

## Abstract

**Background:**

We have recently identified HOP hoemobox (HOPX) as a tumor suppressor gene candidate, characterized by tumor-specific promoter DNA hypermethylation in human cancers, and it can remarkably inhibit tumors’ aggressive phenotypes. In this current study, we for the first time examined methylation level of HOPX and tested the functional relevance in pancreatic cancer (PC).

**Methods:**

Clinical features of HOPX promoter hypermethylation was investigated in 89 PC tissues, and immunohistochemistry was added. We also examined its functional relevance in phenotype assays such as soft agar, proliferation, invasion, and cell cycle analysis.

**Results:**

PC tissues had HOPX gene hypermethylation as compared to the corresponding normal pancreas tissues, and its uniqueness was robust to discriminate tumor from normal tissues (AUC = 0.85, P < 0.0001). Unexpectedly, HOPX was increased in expression in tumor tissues, and immunohistochemistry revealed its predominant expression in the Langerhans islet cells, where HOPX was reduced in expression for PC cells with promoter hypermethylation. HOPX transfectants exhibited G1 arrest with subG1 accumulation, and inhibited tumor forming and invasive ability.

**Conclusion:**

Defective expression of HOPX which is consistent with promoter DNA hypermethylation may explain aggressive phenotype of pancreatic cancer, and intense expression of HOPX in the Langerhans cells may in turn uniquely contribute to pancreatic carcinogenesis.

## Background

Global hypomethylation is often accompanied by dense hypermethylation of the specific promoters in human cancers [1-3]. Promoter hypermethylation results in gene silencing, and such genes have proved to have potent tumor suppressive function and is rather rare [1,4]. We previously developed pharmacologic reversal of epigenetic silencing and uncovered a myriad of transcriptionally repressed genes in human cancers [5,6]. Using this technique, we have identified several unknown tumor suppressor gene candidates, which included HOP homeobox (HOPX) [7,8].

HOPX gene (GeneBank accession number NT 022853), also known as HOP, NECC1, LAGY or OB1, was initially identified as a gene essential for cardiac growth and development [9]. Three spliced transcript variants, HOPX-α, -β, and -γ, encode the same protein, which contains a putative homeodomain motif that acts as an adapter protein to mediate transcription [10]. HOPX expression is ubiquitous in wide arrays of normal tissue, but not in malignant tissues including choriocarcinoma, lung, uterine endometrial, and gastrointestinal (GI) cancers [7,8,11-15]. The inactivation mechanism actually involves promoter methylation in esophageal, endometrial, and gastric cancer [7,8,15]. Also, enforced HOPX expression inhibited tumor growth and RNA interference knockdown of endogenous HOPX restored it [7,8,15]. These findings suggest that the HOPX gene acts as a tumor suppressor gene.

In this study, we for the first time studied methylation level of HOPX gene in PC and added the functional assay to answer the question whether HOPX plays an important role in pancreatic carcinogenesis.

## Methods

### Cell lines and tissue samples

The pancreatic cancer cell lines, PK-8, KLM-1, and NOR-P1 were kindly provided from the Cell Resource Centre for Biomedical Research Institute of Development, Aging and Cancer, Tohoku University (Sendai, Japan). Six other cell lines, PK-59, PK-45 H, PK-45P, MIA Paca2, PANC-1, or the esophageal squamous cell carcinoma (ESCC) cell line TE15 [16] and gastric cancer cell line KatoIII were purchased from RIKEN BioResource Centre (Ibaraki, Japan). All cell lines except MIA Paca2 were maintained in RPMI 1640 Medium (GIBCO, Carlsbad, CA) and MIA Paca2 was maintained in DMEM (GIBCO), containing 10% fetal bovine serum.

Clinical tissue samples were categorized according to TNM classification, 7^th^ edition of the Union Internationale Contre Le Cancer (UICC) and the 6^th^ edition of the Japan Pancreas Society (JPS). The patients’ characteristics were depicted in Additional file 1 Table S1. All tissue samples were collected at the Kitasato University Hospital, and informed consent was obtained. The present study was approved by the Ethics Committee of the Kitasato University.

### Bisulfite treatment of DNA and sequencing analysis

Genomic DNA from homogenized bulky tissues and cell lines was extracted using QIAamp DNA Mini Kit (QIAGEN Sciences, Hilden). Bisulfite treatment was done by using an EpiTect bisulfite kit (QIAGEN) and the DNA was applied to polymerase chain reaction (PCR). PCR primer sequences were designed using DNA sequences converted by bisulfited treatment (Table 1). The PCR products were sequenced using a Big Dye® Terminator v3.1 Cycle Sequencing Kit (Applied Biosystems, Foster City, CA). For the clonsed sequence analysis, the PCR products were inserted into pCR4-TOPO vector using a TOPO TA cloning kit for sequencing (Invitrogen, Carlsbad, CA, USA), selected 15 clones for each sample and then sequenced.

**Table 1:** PCR production and sequence of primers and fluorescent probe

**Method**	**Gene**	**Forward primer (5**^**′**^**>3**^**′**^**)**	**Fluorescent (5**^**′**^**3**^**′**^**)**	**Reverse primer (5**^**′**^**>3**^**′**^**)**
bisulfite sequencing^†^	HOPX-β	TAGTITTGTTTGGAAGAGGGGCG		AACCTCCCCTAAAAACAAACTTAAC
Q-MSP^‡^	HOPX-β	TTTGGAGAGGGTTTTAAAGCG	FAM-CGGAGATAGAAGGTCGTTTATCGGGGAGGTCG-TAMRA	AACAAACTTAACAAATCGCGAA
Q-MSP^‡^	β-actin	TGGTGATGGAGGAGGTTTAGTAAGT	FAM-ACCACCACCCAACACACAATAACAAACACA-TAMRA	AACCAATAAAACCTACTCCTCCCTTAA
RT-PCR^§^	HOPX-α and γ	CAAACCCAGGGCTTGCGCTT		GCGGAGGAGCGAAACAGAGAT
RT-PCR^§^/Q-RT-PCR	HOPX-β	GGTCCCCCTTTCGGGAGGAA		GCGGAGGAGAGAAACAGAGAT
RT-PCR^§^/Q-RT-PCR	HOPX-core	CAGAGGACCAGGTGGAAATCC		GCGGAGGAGAGAAACAGAGAT
RT-PCR^§^/Q-RT-PCR	β-actin	GCTCGTCGTCGACAACGGCTC		CAAACATGATCTGGGTCATCTTCT
PCR for cloning^#^	HOPX	CACCATGTCGGCGGAGACCGCGAGCGG		GTCTGTGACGGATCTGACACTCTG

Abbreviations: Tm, annealing temperature.

^†^: Bisulfite sequencing PCR was done at 95°C for 3 min followed by 40 cycles at 95°C for 1 min, 72°C for 2 min, and final extension at 72°C for 10 min,in a 50 μl reaction volume containing 1 μl treated DNA, 5 μl 10× PCR buffer, 0.2 μm ol/l MgCl_2,_ 0.2 μmol/l each primer and 0.2 μl Platinum® Taq DNA Polymerase.

‡: Q-MSP was done at 95°C for 3 min followed by 45 cycles at 95°C for 20 sec, 60°C for 30 sec, and 72°C for 30 sec, in a 25 μl reaction volume containing 100 ng treated DNA, 300 nmol/l fluorescent probe, and 25 μl iQ^TM^ supermix.

§: RT-PCR was done at 95°C for 3 min followed by 30 cycles at 95°C for 1 min, 60°C for 1 min, and final extension at 72°C for 1 min, and final extension at 72°C for 10 min, in a 50 μl reaction volume containing 1 μl cDNA, 5 μl 10× PCR buffer, 0.2 μmol/l dNTP mixture, 1.5 mmol/l MgCl_2,_ 0.2 μm ol/l each primer and 0.2μl Platinum® Taq DNA Polymerase.

: Q-RT-PCR was done at 95°C for 2 min followed by 40 cycle at 95°C for 15 sec, 60°C for 30 sec, and 72°C for 30 sec, in a 25 μl reaction volume containing 100 ng treated DNA, 300 nmol/l each primer, and 25 μl iQ^TM^PCR Mix.

#: PCR was done at 94°C for 2 min followed by 35 cycles at 94°C for 15 sec, 64.8°C for 15 sec, 68°C for 30 sec, and final extension at 68°C for 7 min, in a 50 μl reaction volume containing 1 μl cDNA, 5 μl 10× Pf×50^TM^PCR Mix, 0.3 μmol/l dNTP mixture, 0.3 μmol/l each primer and 1 μl Pf×50^TM^ DNA Polymerase.

### Quantitative-methylation-specific PCR (Q-MSP)

TaqMan methylation specific PCR (Q-MSP) was carried out using iQ Supermix (Bio-Rad) in triplicate on the iCycler iQ^TM^ Real-Time PCR Detection system (Bio-Rad). PCR conditions and the primer sequences are provided in Table 1. Serial dilutions of bisulfite modified DNA from KatoIII were used as positive control and TE15 as negative control, respectively. The methylation value was defined by a ratio of HOPX-β divided by β-actin and then multiplied by 100, according to the comparative cycle threshold (C_T_) method [17].

### RNA purification and reverse transcriptase-polymerase chain reaction

Total RNA from homogenized bulky tissues and cell lines was extracted using RNeasy Mini Kit (QIAGEN), and reverse-transcribed with a SuperScript III Reverse Transcriptase kit (Invitrogen). Quantitative real time RT-PCR (Q-RT-PCR) for HOPX-β or HOPX-core was performed using iQ^TM^ SYBR Green Supermix (Bio-Rad) in triplicate on the iCycler iQ^TM^ Real-Time PCR Detection system (Bio-Rad), either (Table 1). Relative quantitative analysis adjusted for β-actin was performed according to the C_T_ method [17]. Table 1 depicts sequences of primers/probes and PCR condition.

### Immunoprecipitation and Western blotting

Whole cells lysates were obtained using RIPA buffer (Pierce, Rockford, IL) supplemented with 10 μL/ ml Halt^TM^ Protease Inhibitor Cocktail Kit (Pierce) and Halt^TM^ Phosphatase Inhibitor Cocktail Kit (Pierce). Immunoprecipitation (IP) was performed using Dynabeads Protein G (Dynal Biotech, Oslo, Norway), 1 μg of anti-HOPX mouse IgG_1K_ monoclonal antibody (3D6, Sigma), and 400 μg of each cell lysates. The anti-HOPX mouse IgG_1K_ monoclonal antibody (3D6, dilution of 1:1000, Sigma), anti-HOPX rabbit IgG polyclonal antibody (FL-73, dilution of 1:200, Santa Cruz Biothecnology, Santa Cruz, CA, USA), anti-V5 mouse IgG_2a_ monoclonal antibody (dilution of 1:5000, Invitrogen), and anti-β-actin mouse IgG_2a_ monoclonal antibody (dilution of 1:10000, Sigma) were used for Western blotting (WB) or IP/WB.

### 5-Aza-dC and TSA treatment

Cells (1×10^6^ cells/T-75 flask) were treated with 1 or 5 μM of the demethylating agent 5-aza-2′-deoxycytidine (5-Aza-dC) (Sigma-Aldrich) dissolved in 50% acetic acid or mock-treatment with PBS including the same amount of acetic acid every 24 hrs for 4 days. When combined with the histone deacetylase inhibitor trichostatin A (TSA) (Sigma-Aldrich), 300 nM TSA was added to the medium for the final 24 hrs.

### Immunohistochemistry

Formalin fixed, paraffin-embedded histological sections (3 μm thick) were immunostained using the HOPX antibody (3D6, dilution of 1:200). And immune complexes were detected using the 3,3′-diamino-benzidine tetrahydrochloride (DAB) substrate, as a chromogen for 30 seconds or 2 minutes.

### Plasmid and transfection

A full length cDNA of HOPX was previously isolated and subcloned into pcDNA^TM^3.1D/V5-His-TOPO vector (pcDNA^TM^3.1-HOPX) [8]. The vector with self-ligation was used as a mock control. Plasmid vectors were transfected into 2 pancreatic cancer cell lines (MIA Paca2 and PANC-1) using Lipofectamine 2000 reagent (Invitrogen). Stable clones with HOPX or mock were established by G418 (GIBCO) selection (MIA Paca2, 800 μg/ml; PANC-1, 1200 μg/ml).

### Proliferation assay

Cell proliferation and viability (2×10^3^ cells/well) were measured using the Premix WST-1 Cell Proliferation Assay System (Takara Bio, CO., Tokyo, Japan) in 96-well plates. Experiments were performed in triplicate.

### Invasion assay

Cells were seeded at density of 1× 10^6^ cells/well in the 24-well BD BioCoat Matrigel Invasion Chamber (BD Biosciences Discovery Labware, Bedford, MA) filled with 500 μl DMEM (GIBCO). As a chemoattractant, 10% FBS in 750 μl DMEM (GIBCO) was used for the assay. After incubation for 22 hrs, the membrane of the upper chamber was fixed and stained by Diff-Quick reagent (Sysmex, Kobe, Japan). Invaded cells were counted in for randomly selected sites per membrane.

### Anchorage-independent colony formation assay

Anchorage-independent cell growth was analyzed by plating 0.36% top agarose (Bacto^TM^ Ager, Becton Dickison and Company, Franklin Lakes, NL) containing 1×10^5^ cells on a surface of 0.72% bottom agarose in 6-well plates. Two independent experiments were performed and each experiment was done in triplicate.

### Cell cycle assay

Cells (1× 10^6^ cells/ml) were fixed in 75% ethanol, 5×10^5^ cells stained with propidium iodide (Guava cell cycle reagent, Guava Technologies, Hayward, CA) and the cell cycle assay was carried out using the Guava PCA System. The experiment was performed in triplicate and analyzed using CytoSoft 2.1.5 software (Guava Technologies).

### Statistical analysis

Fisher’s exact test, the chi-square test, Mann-Whitney’s U test or Kruskal-Wallis rank test used for categorical variables, and Student’s t-test was used for continuous variables. Clinicopathological characteristics and follow up data were analyzed in association with 5 year disease specific survival (DSS). The follow up time was calculated from the date of surgery to death. DSS was calculated by Kaplan-Meier method, and survival differences were assessed in the log-rank test. Variables suggested to be prognostic factors on univariable analysis (P < 0.05) were subjected to multivariable analysis using a Cox proportional-hazards regression model. Data is expressed as mean ± standard deviation (SD), and P-value <0.05 was considered to indicate statistical significance. All statistical analyses were conducted with SAS software package (SAS Institute, Cary, NC).

## Results

### Structure of HOPX promoter region

**Figure 1:**
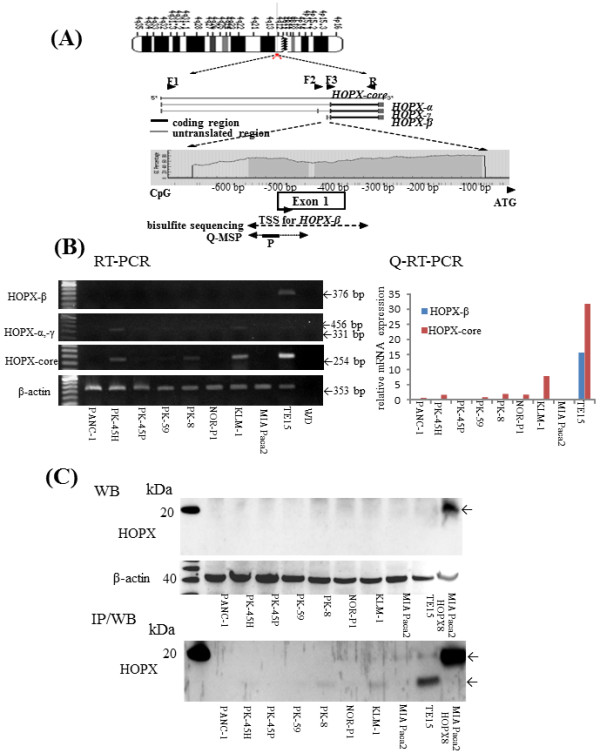
**Analysis of HOPX-β methylation and expression in pancreatic cancer cell lines-1.** (**A**) Schematic diagram of the 3 spliced transcript variants and common transcript core in HOPX (middle panel) and of CpG islands (gray area) in the 5′-flanking region of HOPX gene (bottom panel). Vertical bars indicate the dinucleotides CpG. Arrows indicate the sequences for bisulfite sequencing analysis or Q-MSP, respectively. F1, F2 and F3 represent forward primers for HOPX-α (331 bp) and HOPX-γ (456 bp), HOPX-β (376 bp), and HOPX-core (254 bp) in RT-PCR or Q-RT-PCR; R, common reverse primer; P, probe for Q-MSP; TSS, transcription start site; ATG, translation start codon. (**B**) Expression level of HOPX in PC cell lines was examined by RT-PCR (left panel) and Q-RT-PCR (HOPX-β and core/β-actin x 100, (right panel). (**C**) Expression level of HOPX in PC cell lines was examined by WB (top panel) and IP/WB (bottom panel). Transfectants we performed had the V5 epitope and polyhistidine region in the C-terminal peptide, and so, added approximately 5 kDa to original protein.

Genomic structure of HOPX gene is shown in Figure 1A. HOPX has 3 unique transcript variants with 2 discrete promote regions, and all of the 3 transcripts have the same open reading frame. Among the 3 variants, only HOPX-β harbours promoter CpG islands (promoter B) encompassed by the first exon and intron [8].

### Expression level of HOPX transcripts (core and each transcript-α, β, and γ) and protein in PC cell lines

We first examined expression of HOPX core transcripts, in which PCR primers were shared for all the 3 transcripts (Figure 1A). Among the 8 PC cell lines tested, only KLM-1, PK-8, and PK-45 H cells express slight expression of HOPX core transcript, where HOPX core transcript is consistent with HOPX-α/-γ transcripts. On the other hand, HOPX-β was not detected at mRNA level in any 8 PC cell lines (Figure 1B). WB analysis also revealed that HOPX protein was hardly detected in any PC cell lines. We therefore added experiments of IP/WB (Figure 1C), which could detect very weak but consistent expression of HOPX protein in KLM-1, PK-8, and PK-45 H at 10 kDa [7,9,14]. As HOPX transfectants and positive control TE15 showed considerable expression (Figure 1C), we concluded that HOPX expression showed very little, if any, in PC cells.

### Characteristics of promoter B methylation in PC cell lines

**Figure 2:**
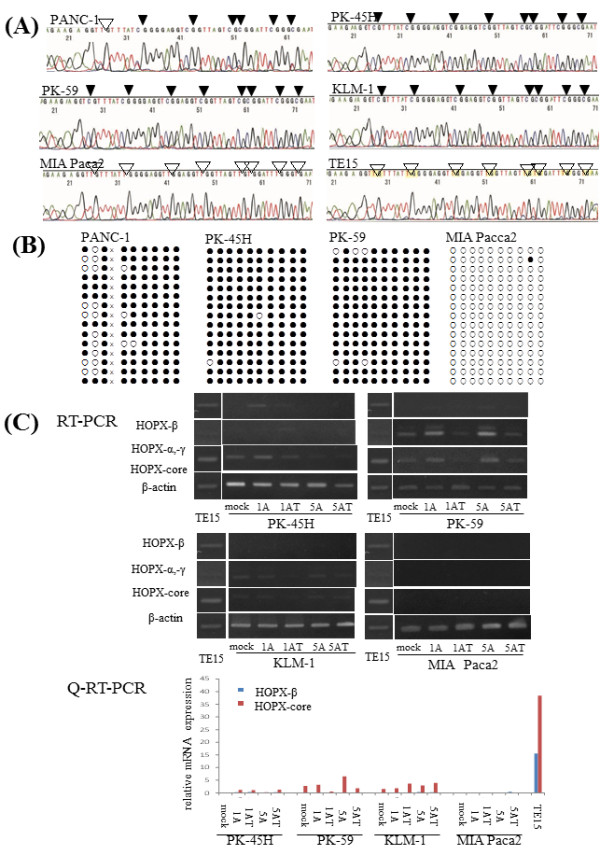
**Analysis of HOPX-β methylation and expression in pancreatic cancer cell lines-2.** (**A**) Representative bisulfite sequencing results in 5 PC cell lines and TE15. Arrowhead indicates dinucleotide CpG. (**B**) Cloned PCR products from PC cell lines. White and black circles denote unmethylated and methylated CpG sites, respectively. X means seven nucleotide deletion; AGGCCGG. (**C**) mRNA expression by RT-PCR (top panel) and Q-RT-PCR (bottom panel) after treatment with the demethylation agent, 5-aza-dC, in the presence or absence of TSA, a histone deacetylase inhibitor. 1A and 5A, 1 and 5 μM 5-aza-dC; T, TSA.

We initially examined the HOPX-β promoter methylation in all the 8 PC cell lines by bisulfite treatment followed by direct sequencing, and promoter B of HOPX gene was proved to be completely methylated in cytosine residues of CpG islands in 7 PC cells except PANC-1 and MIA Paca2 (Figure 2A, B).

In order to demonstrate whether HOPX silencing of PC cells is due to epigenetic abnormalities, demethylating agents such as 5-Aza-dC and/or trichostatin A were added to PC cells, and reactivation of HOPX transcripts was evaluated by RT-PCR and Q-RT-PCR (Figure 2C). Differently from other GI cancers, reactivation was recognized in small portions of PC cells to the small extent (Figure 2C). Reactivation by the most optimal demethylating conditions was only found in PK-45 H and PK-59, suggesting that reduction of HOPX gene could be partially explained by epigenetic alterations of HOPX promoter B. HOPX absent expression in MIA Paca2 was also confirmed even after epigenetic reversion. We could have therefore postulated homozygous deletion to explain this absent expression. However, DNA could be amplified for the promoter regions of HOPX-β, and actual cloned sequence largely showed unmethylation in MIA Paca2 (Figure 2B). Other cancer cell lines also showed very little expression of HOPX-β, so constitutive transcription signal to activate HOPX-β expression was defective in PC cell lines. However, this result does not represent meaningless significance of HOPX methylation, and we continued methylation analysis in primary PC tissues.

### Expression of HOPX transcripts and protein in PC tissues and the corresponding normal pancreas tissues

**Figure 3:**
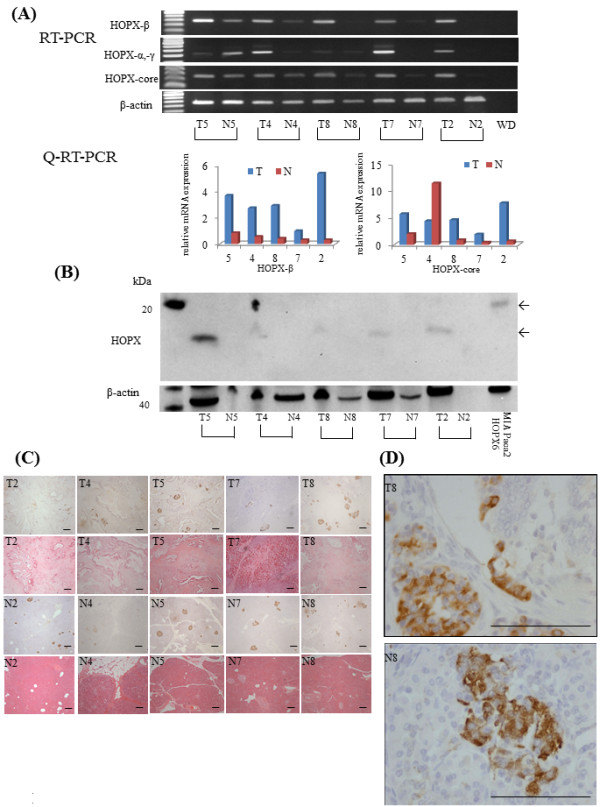
**HOPX expression status in PC.** (**A**) Expression level of HOPX in PC was tested in RT-PCR (top panel) and Q-RT-PCR (bottom panel). T, primary tumor; N, corresponding pancreatic tissue. (**B**) Expression level of HOPX in PC was examined by western blotting. (**C**) Immunohistochemical staining for HOPX in primary tumor (top panel) and normal tissue (bottom panel), with hematoxylin eosin staining (original magnification, X40). These immunohistochemical stainings were performed by short term exposure of DAB. (**D**) In this condition, islet cells only stained (original magnification, X400). scale Bars, 100 μm.

We first examined the expression status of HOPX transcripts for both the primary tumors and the corresponding pancreas tissues in the 5 consecutive advanced PC patients by both semi-quantitative RT-PCR and Q-RT-PCR. As a result, HOPX-β transcripts were rather robustly over-expressed in the primary PC tissues as compared to the corresponding normal tissues (Figure 3A). WB also showed HOPX protein over-expression in tumor tissues as compared to in the corresponding normal tissues (Figure 3B).

In order to confirm predominant localization of HOPX protein in primary PC, we then performed immunohistochemistry (Figure 3C, D). Surprisingly, HOPX was strongly immunostained almost exclusively for pancreatic islet cells by short term exposure (30 seconds) of DAB. Neither cancer cells nor normal pancreatic components such as acinar and ductal cells showed staining of HOPX (Figure 3D). On the other hand, islet cells in normal pancreas also showed considerable immunostaining of HOPX (Figure 3D). These findings suggested that predominant expression of HOPX transcripts and protein in primary PC represents those of islet cells.

**Figure 4:**
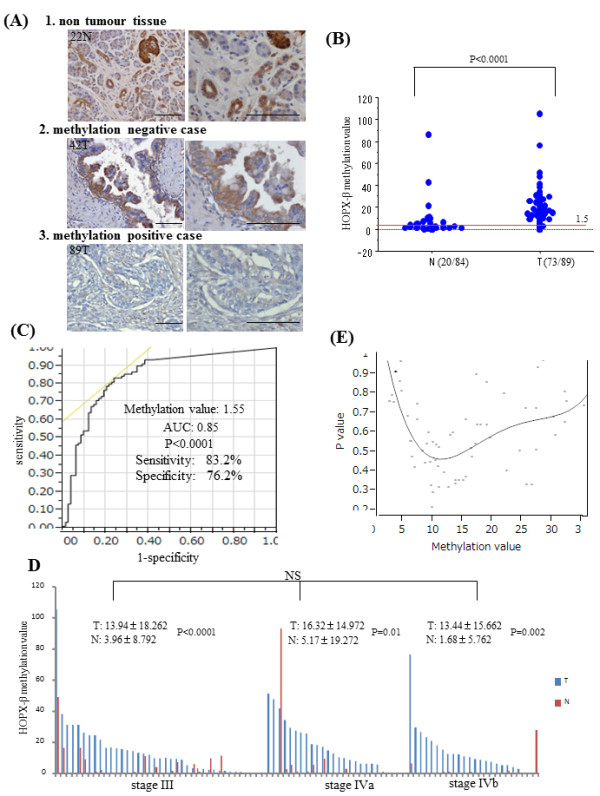
**Immunohistochemistry and Quantitative methylation analysis in 89 samples.** (**A**) Representative immunohistochemical staining for HOPX in normal tissues and primary tumor with or without HOPX-β hypermethylation (original magnification, left X200, right X400). scale Bars, 100 μm. (**B**) Frequency of HOPX-β hypermethylation by Q-MSP. Dashed line indicates the optimal cut-off value (1.5). (**C**) ROC curve of HOPX-β methylation for detection of PC. Area under the curve (AUC) represents the accuracy in discriminating normal from tumor in term of sensitivity and specificity (P < 0.0001). (**D**) Methylation value of HOPX-β in JPS stage III, IVa and IVb. Data are expressed as mean ± SD. (**E**) Identification of an optimal cut-off value for the prognosis using the log rank prognostic analysis.

Instead of intense immunostaining of pancreas islet cells, pancreatic duct and a portion of acinar cells were also immunostained by intermediate exposure (2 minutes) of DAB (Figure 4A). The cellular localization of HOPX existed mainly in cytoplasm. Under such conditions, we investigated the IHC staining of HOPX for 11 cases with high methylation value and 9 cases with low methylation value, respectively. In high methylation value tissues, absent expression of HOPX was confirmed despite frequent inclusion of heterogeneity. However, 9 samples with low methylation value exhibited relatively strong HOPX expression, while only one sample showed negative expression (Figure 4A). These results indicated that expression of HOPX protein was associated with promoter hypermethylation.

Using the same primers and probes in gastric cancer study [8], we examined both 89 primary PC tissues and 84 corresponding non tumor tissues by Q-MSP analysis (Figure 4B). The most optimal cut-off value was calculated for 1.5 from a receiver-operator characteristic (ROC) analysis in order to maximize both sensitivity and specificity of PC detection (Figure 4C), where sensitivity was 83.2%, and specificity was 76.2%.

The overall methylation value detected in primary PC tissue (14.50 ± 16.53) was significantly higher than that in the non tumoral tissues (3.64 ± 12.02) (P < 0.0001) (Figure 4B). In addition, the methylation values within primary PC tissues were significantly higher than those within non tumor tissues in individual patients, whereas methylation values did not significantly differ in each stage (Figure 4D).

We further investigated whether the HOPX-β methylation value was able to predict patient’s outcomes. Log-rank plot analysis [18] showed that any cut-off value could not represent prognostic stratification in PC (Figure 4E). We preliminarily analyzed the correlation between HOPX-β hypermethylation and the clinicopathological parameters, but none of any clinicopathological variables was associated with methylation status of HOPX-β (Additional file 2 Table S2).

### HOPX stable transfectants caused suppression of aggressive PC cell phenotypes

**Figure 5:**
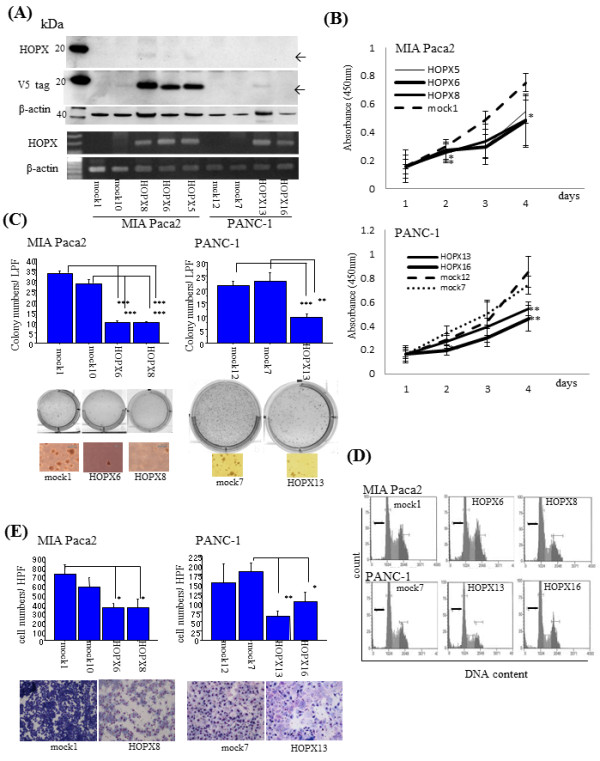
**Functional analysis of HOPX in PC cells.** (**A**) HOPX expression level in HOPX expressing stable cell lines was determined by mRNA expression (lower panel) and protein expression (upper panel). HOPX protein was detected by WB with HOPX antibody (3D6) and the flag V5 antibody. β-actin was shown as a loading control. (**B**) Proliferation assay was performed for 5 days. Data are shown as absorbance at 450 nm. error bars, SD. (**C**) Anchorage-independent colony formation assay was performed. After 3 weeks of cell culture, colonies were counted and photographed at 40× magnification under a microscope. Colonies were also visualized by ethidium bromide staining. error bars, SD. (**D**) Image of cell cycle assay. Thick black bars, subG1 phase. (**E**) Matrigel invasion assay. After fixation and staining, invading cells were photographed and counted at 100x magnification. error bars, SD.

Two cell lines of pancreatic adenocarcinoma such as PANC-1 and MIA Paca2 cells were transfected with pcDNA^TM^3.1-HOPX with V5-tagged and established stable HOPX-expressing cell lines. In the HOPX stable cell lines, exogenous mRNA expression level in cells with the most abundant expression was comparable to physiological expression level in human PC tissues. HOPX protein was confirmed by 3D6 antibody and anti-V5 antibody. Exogenously expressed tagged HOPX was detected as approximately 15 kDa which is consistent with mRNA levels (Figure 5A).

HOPX transfectants showed both less viability by WST assay (Figure 5B) and remarkable reduction of colonies in soft agar (Figure 5C) as compared with mock cells. Moreover, we found considerable suppression of invasion activity in HOPX-expressing cells by Matrigel invasion assay (Figure 5E). Cell cycle analysis further revealed that HOPX increased fractions of both subG1 fraction and G0/G1, accompanied by decreased fraction of both S and G2/M, indicating that both G1 arrest and apoptotic sensitivity may be at least partially involved in tumor suppressive traits of HOPX-expressing cell (Figure 5D and Additional file 3 Table S3).

## Discussion

We have recently identified HOPX as genes specifically methylated in human cancers [7,8] after developing algorithm utilizing pharmacological unmasking microarray (PUM) [5,6]. Among the identified candidates of TSGs, HOPX is of particular interest in terms of methylation and functional involvement in tumor aggressiveness. Other groups also recapitulated the similar finding that HOPX promoter DNA is hypermethylated specifically in endometrial cancer [15]. In this present study, we for the first time added pancreatic cancer to the list of organs in which HOPX is involved in carcinogenesis.

HOPX harbors 2 discrete promoter regions, promoter A and promoter B. Promoter B has CpG islands, while promoter A does not have them, and cancer-specific hypermethylation is recognized in the promoter B in primary PC tissues as well as other GI cancers [7,8]. Such independent regulation of the discrete promoter regions was reported in other critical methylation genes such as RASSF1 [19], and possession of the complex promoter regions may indicate their functional importance in biological relevance. On the other hand, epigenetic reactivation of HOPX gene expression was much less than expected in PC cell lines as compared to other GI cancer cell lines. Allowing for actual expression in primary cancer tissues, constitutive HOPX expression signal was derived from carcinoma-stroma interaction in primary PC cells.

Pancreatic cancer is a ductal carcinoma, however it is controversial which normal components (ductal cells, acinar cells, or islet cells) of the pancreatic tissues are precursor cells for PC [20,21]. Pour et al. proved that transplantation of islets into the submandibular gland of Syrian golden hamsters followed by treating with nitrosamine N-nitrosobis-(2-oxopropyl)amin (BOP), a carcinogen for PC led to the development of ductal pancreatic adenocarcinoma in this site, while PC did not occurred after transplanting ductal and acinar cells into this gland [22]. Schmied et al. has also insisted that islet cells contribute to pancreatic carcinogenesis in an animal model and disease exploration [23,24]. In mice with hamster islets implanted in the splenic lobe of the mouse pancreases, pancreatic ductal adenocarcinomas developed in the implanted animals, but not in control mice, after BOP treatment [25]. These findings strongly supported the hypothesis that PC is generated from islet cell origin. In this current study, we for the first time revealed that islet cells expressed abundant HOPX protein in primary PC tissues as well as the normal pancreas. It is intriguing hypothesis that cancer cell with low expression of HOPX is derived from islet cells which constitutively express abundant HOPX, and that promoter DNA hypermethylation is causative for gene silencing.

Clinical findings also supported hypothesis that the islet cell is alternatively involved in PC carcinogenesis [23], in which remarkable alteration of quality of islet cells was observed in primary PC tissues. Ten out of the 14 cancer specimens showed a significant loss of beta cells (P < 0.005) and eight of them also showed a significant increase of alpha cells (P < 0.005), all of them from hyperglycemic patients. Most affected islets were found within zone 1 (intratumoral) and zone 2 (peritumoral), to a lesser extent in zone 3 (acini close to tumor) and none in zone 4 (acini remote from tumor). The incidence of 72% with alteration of islets in their material correlates with the frequency of abnormal glucose levels in human pancreatic cancer patients. In our study, HOPX is remarkably increased in primary PC tissues, and it was predominantly expressed in the islet cells. These findings suggested that alteration of HOPX expression in the islet cells may explain the link of PC to diabetes mellitus, and this mechanistic possibility should be paid attention in the next future, as oncogenic role of islet cells remains elusive during PC carcinogenesis.

HOPX actually suppressed tumor aggressiveness of PC cells (PANC-1 and MIA Paca2). WST assay showed that HOPX suppressed cell viability putatively representing cell proliferation ability. In cell cycle analysis, HOPX increased subG1 and G0/G1 phases, representing apoptotic induction and inhibition of DNA synthesis, suggesting that cell cycle abnormalities may be linked to cell viability. More importantly, HOPX could inhibit tumor-forming ability in soft agar, which is supposed to represent metastatic trait of tumor cells [26]. Interestingly, HOPX has been demonstrated to suppress tumorigenesis in soft agar in ESCC and gastric cancer as well as pancreatic cancer, hence anchorage independent growth suppression is the common feature of HOPX expression in human cancers. Finally, HOPX also affects Matrigel invasion less than other phenotypes in PC. These findings may directly show gene silencing of HOPX involved in PC aggressiveness.

Such tumor suppressive effects as shown in Figure 5 might include artifact effect, because expression level of HOPX protein in transfectants may not correspond to the physiological level of the originated normal cell, if precursor cells of the PC were ductal or acinar cells. On the other hand, HOPX expression level of the islet cells reached similar level of the transfectants in our current study. More importantly, the level of expression in the transfectants of the current study was comparable with those of normal mucosa of other tissues such as gastric [8] and colorectal mucosa [27]. As compared to such common solid tumors, PC exhibited uniquely dismal prognosis, which is consistent with low expression of HOPX in PC. As constitutive expression of HOPX in human cancer cell lines including PC cell lines was infrequently found, RNA knockdown experiments was impossible to verify the endogenous role of HOPX in human pancreatic cancer cells, however we previously investigated RNA knockdown effects of HOPX by using esophageal cancer cells, TE15 that is a rare control cell which constitutively expressed HOPX [7,8], and tumor suppressive role was confirmed. DNA hypermethylation of HOPX with gene silencing is therefore likely to affect PC phenotypes as in other cancers. On the other hand, there were some limitations of the conclusions that can be made based on our functional assay. In MIA Paca2 cells, HOPX was unlikely to be inactivated by methylation, and transfected HOPX protein of PANC-1 cells was expressed relatively weakly. Hence, our conclusion on tumor suppressive role of HOPX on PC was based largely on epigenetic characteristics in primary PC, and results of PC cell lines remained supplementary. We would like to know more specific and definitive conclusions as to these concerns in the near future.

HOPX affects gene transcription through recruitment of HAT and/or HDAC activity for specific transcriptional factors [28-30]. Yeast two hybrid identified enhance of polycomb-1 (Epc-1), a critical component of NuA4 HAT complex, as a binding partner of HOPX, and augments transcription of heart differentiation genes. Interestingly, Epc1 was demonstrated to be associated with EZH2 which is required for cellular proliferation, E2F6-PcG complex (E2F6-EPC1) that interacts with EZH2 and may regulate genes required for cell cycle progression [31,32]. Thus, HOPX may therefore affect critical process of chromatin conformation change to affect expression of onco-molecules.

Collectively, we found that HOPX methylation is a very frequent and cancer specific event in PC development. We further elucidated that HOPX is a putative tumor suppressor gene critical for tumor aggressiveness in PC. We are also interested in alternate aspects of HOPX in terms of a role in islet cells. We must confirm more detailed mechanism involved in remarkable phenotype alteration by HOPX abnormalities in PC in future study.

## Conclusions

Defective expression of HOPX which is consistent with CpG islands promoter DNA hypermethylation may explain aggressive phenotype of pancreatic cancer, and intense expression of HOPX in the Langerhans islet cells may in turn uniquely contribute to pancreatic carcinogenesis.

## Abbreviations

HOPX: Homeodomain-only protein; PC: Pancreatic cancer; Q-MSP: TaqMan methylation specific polymerase chain reaction; Q-RT-PCR: Quantitative reverse transcriptase-polymerase chain reaction; UICC: The union internationale contre le cancer; JPS: Japan pancreas society; ROC curve: Receiver-operator characteristic curve; SD: Standard deviation; DSS: Disease specific survival.

## Competing interests

There is no conflict of interest that could be perceived as prejudicing the impartiality of the research reported.

## Authors’ contributions

MW conceived of the study, performed the study, drafted the manuscript and participated in coordination. KY participated in coordination and assisted in editing of manuscript. HK, AO, HK, HN, KN, and AE helped in the collection and analysis of clinical data. MW participated in coordination. All authors read and approved the final manuscript.

## Pre-publication history

The pre-publication history for this paper can be accessed here:

http://www.biomedcentral.com/1471-2407/12/397/prepub

## Supplementary Material

Additional file 1 **Table S1.** Characteristics and prognostic analysis in 89 patients with pancreatic cancer. Click here for file

Additional file 2 **Table S2.** Correlation analysis between HOPX-β methylation status andclinicopathological variables (n= 89). Click here for file

Additional file 3 **Table S3.** Distribution of cell cycle phase. Click here for file
