# Supplementary material for: Cancer specific promoter CpG Islands hypermethylation of HOP homeobox (HOPX) gene and its potential tumor suppressive role in pancreatic carcinogenesis
Source: BMC Cancer. 2012 Sep 7;12:397. doi: 10.1186/1471-2407-12-397 (PMC3488580; doi:10.1186/1471-2407-12-397)
Supplement: Additional file 1 — Table S1. Characteristics and prognostic analysis in 89 patients with pancreatic cancer. [file 1471-2407-12-397-S1.pdf]

Supplemental Table 1. Characteristics and prognostic analysis in 89 patients with pancreatic cancer

Variables	Number	MST (month)	5 year DSS Percentage(%)	univariable analysis	multivariable analysis†	
				P Value[a]	HR (95%CI)	P Value[b]
**Age(years)**						
**<65/**>65	51/38	11.1/18.6	15.1/20.2	0.1	-	-
**Gender**						
Male/**Female**	46/43	11.3/16.4	12.3/22.1	0.13	-	-
**Methylation value of HOPX-β**						
<1.55/**>1.55**	15/74	16.4/12.1	20.0/6.7	0.4	-	-
**preoperative serum CA19-9**						
<37 U/**ml/>37 U/ml**	23/66	-/10.4	53.0/4.6	<0.0001	3.31 (1.48-7.41)	0.0016
**Location**						
**head**	68	11.3	17.9	0.3		
**body**	15	29.5	16.3			
**tail**	6	16.33	0.0			
**Growth pattern**						
nodular/**other**	35/54	18.6/10.4	19.7/16.0	0.05	-	-
**histology**						
**well**	37	18.8	22.5	0.02	reference	0.028
**moderately**	34	11.1	13.5		1.57 (0.85-2.88)	
**poor**	18	6.5	12.5		2.74 (1.32-5.68)	
**tumor size (cm)**						
**<2**	13	39.1	40.0	0.2	-	-
**2<ts<4**	61	12	14.4			
**4<ts<6**	13	14.8	18.2			
**<6**	2	11.5	0.0			
**ND factor**						
ND<10/**ND10**	50/39	16.4/9.5	26.3/7.2	0.01	0.94 (0.52-1.70)	0.85
**pancreatic cut end margin (PCM)**						
negative/**positive**	74/14	16.0/12.1	21.5/0.0	0.1	-	-
**bile duct cut end margin (BCM)**						
negative/**positive**	70/1	11.9/14.8	20.2/9.4	0.9	-	-
**dissected pancreatic tissue margin (DPM)**						
negative/**positive**	48/41	19.7/8.9	33.3/0.0	<0.0001	2.82 (1.17-6.80)	0.013
**residual tumor (R factor)**						
**0**	35	23.5	38.3	0.0002	reference	0.45
**1**	40	11.9	7.2		0.77 (0.30-1.92)	
**2**	14	7.1	0.0		1.16 (0.35-3.77)	
**stage (JPS)**						
**III**	39	23.5	28.3	<0.0001	reference	0.0085
**IVa**	26	12.1	19.1		2.06 (1.00-4.20)	
**IVb**	24	7.1	0.0		3.16 (1.48-6.73)	
**T factor (JPS)**						
**1**	1	-	100.0	0.006	-	-
**2**	3	10	0.0			
**3**	58	17.4	22.5			
**4**	27	8.9	9.2			
**lymph node metastasis (N) (JPS)**					-	-
**0**	23	18.6	35.5	0.01		
**1**	34	17	16.8			
**2**	19	11.1	13.4			
**3**	13	7.2	0.0			
**distant metastasis (M) (JPS)**						
absence/**presence**	82/7	16.3/7.0	18.6/0.0	0.0003	-	-
**stage (UICC)**						
**IB**	1	-	100.0	<0.0001	-	-
**IIA**	21	18.6	33.6			
**IIB**	46	17.3	17.8			
**III**	3	3.6	0.0			
**IV**	18	7.2	0.0			
**T factor (UICC)**						
**1**	3	26.5	0.0	<0.0001	-	-
**2**	7	18.2	28.6			
**3**	78	14.8	17.0			
**4**	1	2.2	0.0			
**lymph node metastasis (N) (UICC)**						
**0/1**	23/66	18.6/11.3	35.5/12.2	0.02		
**distant metastasis (M) (UICC)**						
absence/**presence**	71/18	17.3/7.2	22.1/0.0	<0.0001	-	-
**operation**						
**PD**	67	11.3	18.7	0.3	-	-
**DP**	20	18.8	11.1			
**TP**	2	-	100.0			
**lymph node dissection (D)**						
**0,1**	11	18.4	55.5	0.3	-	-
**2**	32	16.3	25.3			
**2+α**	40	11.1	17.0			
**3**	6	9.5	0.0			
**post operative therapy (including adjuvant therapy)**						
absence/**presence**	32/57	11.9/16.4	8.4/20.9	0.1	-	-
**Methylation value of HOPX-β**						
<3.6/**>3.6**	23/66	16.4/12.1	19.2/9.6	0.9	-	-

Abbreviations: DSS disease-specific survival; MST median survival time; HR hazard ratio; CI confidence interval

[a] **log-rank test**

[b] **significant based on Cox's proportional hazard model**

† **Multivariable analysis was indicated excluding constructing factors of JPS staging system, and UICC staging system was not adopted due to divide.**
