# Supplementary material for: Cancer specific promoter CpG Islands hypermethylation of HOP homeobox (HOPX) gene and its potential tumor suppressive role in pancreatic carcinogenesis
Source: BMC Cancer. 2012 Sep 7;12:397. doi: 10.1186/1471-2407-12-397 (PMC3488580; doi:10.1186/1471-2407-12-397)
Supplement: Additional file 2 — Table S2. Correlation analysis between HOPX-β methylation status andclinicopathological variables (n= 89). [file 1471-2407-12-397-S2.pdf]

Supplemental Table 2. Correlation analysis between HOPX-β methylation status and clinicopathological variables (n= 89)

Variables	Total number	HOPX-β hypermethylation			
		negativity	positivity	positive rate (%)	P value
**Age(years)**					
**<65**	51	12	39	76	0.28
**≥65**	38	3	35	92	
**Gender**					
**Male**	46	7	39	85	0.66
**Female**	43	8	35	81	
**preoperative serum CA19-9**					
**<37 U/ml**	23	2	21	91	0.33
**≥37 U/ml**	66	13	53	80	
**Location**					
**head**	68	12	56	82	0.81*
**body**	15	2	13	81	
**tail**	6	1	5	83	
**Growth pattern**					
**nodular**	35	5	30	86	0.6
**other**	54	10	44	81	
**histology**					
**well**	37	8	29	78	0.45*
**moderately**	34	5	29	85	
**poor**	18	2	16	89	
**tumor size (cm)**					
**≤2**	13	3	10	77	0.16*
**2<ts≤4**	61	12	49	80	
**4<ts≤6**	13	0	13	100	
**<6**	2	0	2	100	
**ND factor**					
**ND<10**	50	8	42	84	0.8
**ND10**	39	7	32	82	
**pancreatic cut end margin (PCM)**					
**absence**	75	13	62	83	>0.99
**presence**	14	2	12	86	
**bile duct cut end margin (BCM)**					
**absence**	70	12	58	83	>0.99
**presence**	1	0	1	100	
**dissected pancreatic tissue margin (DPM)**					
**absence**	48	7	41	85	0.53
**presence**	41	8	33	80	
**residual tumor (R factor)**					
**0**	35	3	32	91	0.27*
**1**	40	10	30	75	
**2**	14	2	12	86	
**stage (JPS)**					
**III**	39	7	32	82	0.66*
**IVa**	26	5	21	81	
**IVb**	24	3	21	88	
**T factor (JPS)**					
**1**	1	0	1	100	0.97*
**2**	3	0	3	100	
**3**	58	11	47	81	
**4**	27	4	23	85	
**lymph node metastasis (N) (JPS)**					
**0**	23	4	19	83	0.71*
**1**	34	6	28	82	
**2**	19	4	15	79	
**3**	13	1	12	92	
**distant metastasis (M) (JPS)**					
**absence**	82	14	68	83	>0.99
**presence**	7	1	6	86	
**stage (UICC)**					
**IB**	1	0	1	100	0.53*
**IIA**	21	4	17	81	
**IIB**	46	9	37	80	
**III**	3	0	3	100	
**IV**	18	2	16	89	
**T factor (UICC)**					
**1**	3	1	2	67	0.8*
**2**	7	1	6	86	
**3**	78	13	65	83	
**4**	1	0	1	100	
**lymph node metastasis (N) (UICC)**					
**0**	23	4	19	83	>0.99
**1**	66	11	55	83	
**distant metastasis (M) (UICC)**					
**absence**	71	13	58	82	0.72
**presence**	18	2	16	89	
**operation**					
**PD**	67	12	55	82	0.77*
**DP**	20	3	17	85	
**TP**	2	0	2	100	
**lymph node dissection (D)**					
**0,1**	11	2	9	82	0.19*
**2**	32	8	24	75	
**2+α**	40	4	36	90	
**3**	6	1	5	83	
**post operative therapy (including adjuvant therapy)**					
**absence**	32	4	28	88	0.55
**presence**	57	11	46	81	

* Kruskal-Wallis rank test, the remaining variables: Fisher's exact test or chi-square test
