# Supplementary material for: Cancer specific promoter CpG Islands hypermethylation of HOP homeobox (HOPX) gene and its potential tumor suppressive role in pancreatic carcinogenesis
Source: BMC Cancer. 2012 Sep 7;12:397. doi: 10.1186/1471-2407-12-397 (PMC3488580; doi:10.1186/1471-2407-12-397)
Supplement: Additional file 3 — Table S3. Distribution of cell cycle phase. [file 1471-2407-12-397-S3.pdf]

Supplemental Table 3. Distribution of cell cycle phase

MIA Paca2	subG1(%)	G0/G1(%)	S (%)	G2/M (%)
mock1	0.69±0.07	46.23.48±32.32	25.04±1.62	32.26±1.26
mock10	1.6±0.6	41.24±0.52	24.9±2.37	28.05±2.65
HOPX8	0.93±0.22	72.07±4.29**,**	13.62±0.45**,***	13.37±4.38**,**
HOPX6	2.32±0.29****	38.82±0.15*,**	25.3±0.44	33.57±0.56
HOPX5	2.16±0.22**	38.89±5.39	26.35±1.26	32.6±5.04

PANC-1	subG1(%)	G0/G1(%)	S (%)	G2/M (%)
mock12	1.54±0.17	62.1±0.3	17.44±0.2	18.9±0.387
mock7	0.72±0.09	58.05±0.7	19.29±0.49	21.92±0.35
HOPX13	3.66±0.19****,****	59.5±0.56**,*	10.89±0.27****,****	25.92±0.79***,*
HOPX16	1.17±0.19	62.76±2.55*	14.2±0.61****,****	21.85±0.45*
